# Complete and partial forms of X-linked MCTS1 deficiency in patients with mycobacterial disease

**DOI:** 10.70962/jhi.20250073

**Published:** 2026-01-30

**Authors:** Qinhua Zhou, Ivan Bagarić, Fabian Komma, Chandhana Prakash, Hassan Abolhassani, Zahra Chavoshzadeh, Lulu Tsao, Taja Vatovec, Camille Soudée, Jérémie Rosain, Daniel J. Minter, Conny Lu, Nashat Al-Sukaiti, Bingbing Wu, Jinqiao Sun, Qian Zhang, Jean-Laurent Casanova, Qiang Pan-Hammarström, Alice Y. Chan, Tariq Al-Farsi, Xiaochuan Wang, Jacinta Bustamante, Jonathan Bohlen

**Affiliations:** 1 Department of Clinical Immunology, National Children’s Medical Center, Children’s Hospital of Fudan University, Shanghai, China; 2 Gene Center and Department of Biochemistry, Ludwig-Maximilians-Universität, Munich, Germany; 3 Imagine Institute, Paris Cité University, Paris, France; 4 Laboratory of Human Genetics of Infectious Diseases, Necker Branch, Inserm U1163, Necker Hospital for Sick Children, Paris, France; 5 Division of Immunology, Department of Medical Biochemistry and Biophysics, Karolinska Institutet, Stockholm, Sweden; 6 Research Center for Immunodeficiencies, Pediatrics Center of Excellence, Children’s Medical Center, Tehran University of Medical Sciences, Tehran, Iran; 7 Department of Immunology and Allergy, Mofid Children Hospital, Shahid Beheshti University of Medical Sciences, Immunology and Allergy Department, Tehran, Iran; 8 Division of Pulmonary, Department of Medicine, Critical Care, Allergy and Sleep Medicine, University of California San Francisco Medical Center, San Francisco, CA, USA; 9 Study Center for Primary Immunodeficiencies, Necker Hospital for Sick Children, Asistance Publique-Hopitaux de Paris (AP-HP), Paris, France; 10 St. Giles Laboratory of Human Genetics of Infectious Diseases, Rockefeller Branch, The Rockefeller University, New York, NY, USA; 11 Division of Infectious Diseases, Department of Medicine, University of California San Francisco, San Francisco, CA, USA; 12 Department of Pediatrics, Pediatric Allergy and Clinical Immunology, The Royal Hospital, Muscat, Oman; 13 Division of Allergy, Department of Pediatrics, Immunology and Bone Marrow Transplant, University of California San Francisco, USA; 14 National Children’s Medical Center, Center for Molecular Medicine, Children’s Hospital of Fudan University, Shanghai, China; 15 Howard Hughes Medical Institute, New York, NY, USA; 16 Department of Pediatrics, Necker Hospital for Sick Children, AP-HP, Paris, France; 17 Shanghai Institute of Infectious Disease and Biosecurity, Shanghai, China; 18 Department of Pediatrics, Dr. von Hauner Childrens Hospital, LMU Klinikum, Munich, Germany; 19 German Center for Child and Adolescent Health, Göttingen, Germany; 20 German Center for Child and Adolescent Health, Munich Site, Munich, Germany.

## Abstract

X-linked recessive (XR) complete MCTS1 deficiency underlies Mendelian susceptibility to mycobacterial disease (MSMD) in patients with bacille Calmette-Guérin (BCG) disease. We investigated the genotypic and phenotypic landscape of four new unrelated families from four distinct countries. Three patients had adverse reactions to the BCG vaccine, whereas another patient was not vaccinated with BCG and had an infection with *Mycobacterium abscessus* at 16 years of age. Whole-exome sequencing of the probands revealed hemizygosity for rare germline *MCTS1* variants. In addition to a previously reported loss-of-expression (LOE) and loss-of-function (LOF) variant, we identified three new *MCTS1* variants. The p.L170* and E60Kfs5* variants are LOF, whereas p.W175* is hypomorphic when overexpressed. Thus, we report four new MSMD patients with complete or partial forms of XR MCTS1 deficiency, including three patients with newly discovered genotypes. A diagnosis of partial or complete XR MCTS1 deficiency should be considered in boys and men with MSMD displaying mycobacterial infection.

## Teaser

X-linked MCTS1 deficiency causes susceptibility to mycobacterial disease. Four new patients carried three previously undescribed truncating or loss-of-expression variants, presenting with BCG disease or *M. abscessus* infection. Functional testing showed complete or partial loss of MCTS1 activity, expanding the clinical and genetic spectrum of this disorder.

## Introduction

Mendelian susceptibility to mycobacterial disease (MSMD) is a rare, genetically conferred group of disorders that predispose to clinical infectious disease caused by weakly virulent mycobacteria, such as the *Mycobacterium bovis* strain from the Bacillus Calmette-Guerin (BCG) vaccine and environmental mycobacteria (EM) (1, 2). Interferon-gamma (IFN-γ), a macrophage-activating factor (3), is at the heart of antimycobacterial immunity. Variants of 22 genes underlie 47 different genetic etiologies of MSMD (4). These variants impair the production of IFN-γ (*IFNG*, *IL12B*, *IL12RB1*, *IL12RB2*, *IL23R*, *ISG15, RORC, TBX21*, *TYK2*, and *USP18)*, the response to IFN-γ (*CYBB*, *IFNGR1*, *IFNGR2*, *STAT1*, and *JAK1*), or both (*IRF8*, *IRF1*, *NEMO*, and *SPPL2A*) (4, 5). MSMD can be explained by insufficient peripheral blood monocyte migration to the tissues in CCR2-deficient patients (6). The mechanistic connection between *ZNFX1* and MSMD remains unknown (7). In this context, we recently reported X-linked recessive (XR) complete MCTS1 deficiency as a cause of MSMD (8). The clinical characteristics of the affected patients included disseminated BCG disease (BCG-osis) in the absence of other infectious diseases in all but one individual. This child had not received the BCG vaccine at birth and was only 2 years old at the most recent follow-up visit (8). All the five reported deleterious variants were loss-of-expression (LOE) and complete loss-of-function (LOF). The variants were detected in five families from Iran (two families), Finland, China, and Saudi Arabia. All the detected leukocyte subsets developed normally in the patients. MCTS1 promotes JAK2 translation by reinitiating translation in the JAK2 5′UTR. Adequate levels of JAK2 expression are required for IFN-γ production in response to IL-23 by innate-like adaptive T cells, such as Vδ2^+^ γδ T cells and mucosa-associated innate-like T cells (8). We investigated the allelic and clinical spectrum of MCTS1 deficiency in more detail by searching for additional patients with rare or private *MCTS1* variants.

## Results

### Four boys from four unrelated kindreds with mycobacterial disease

**Figure 1.**
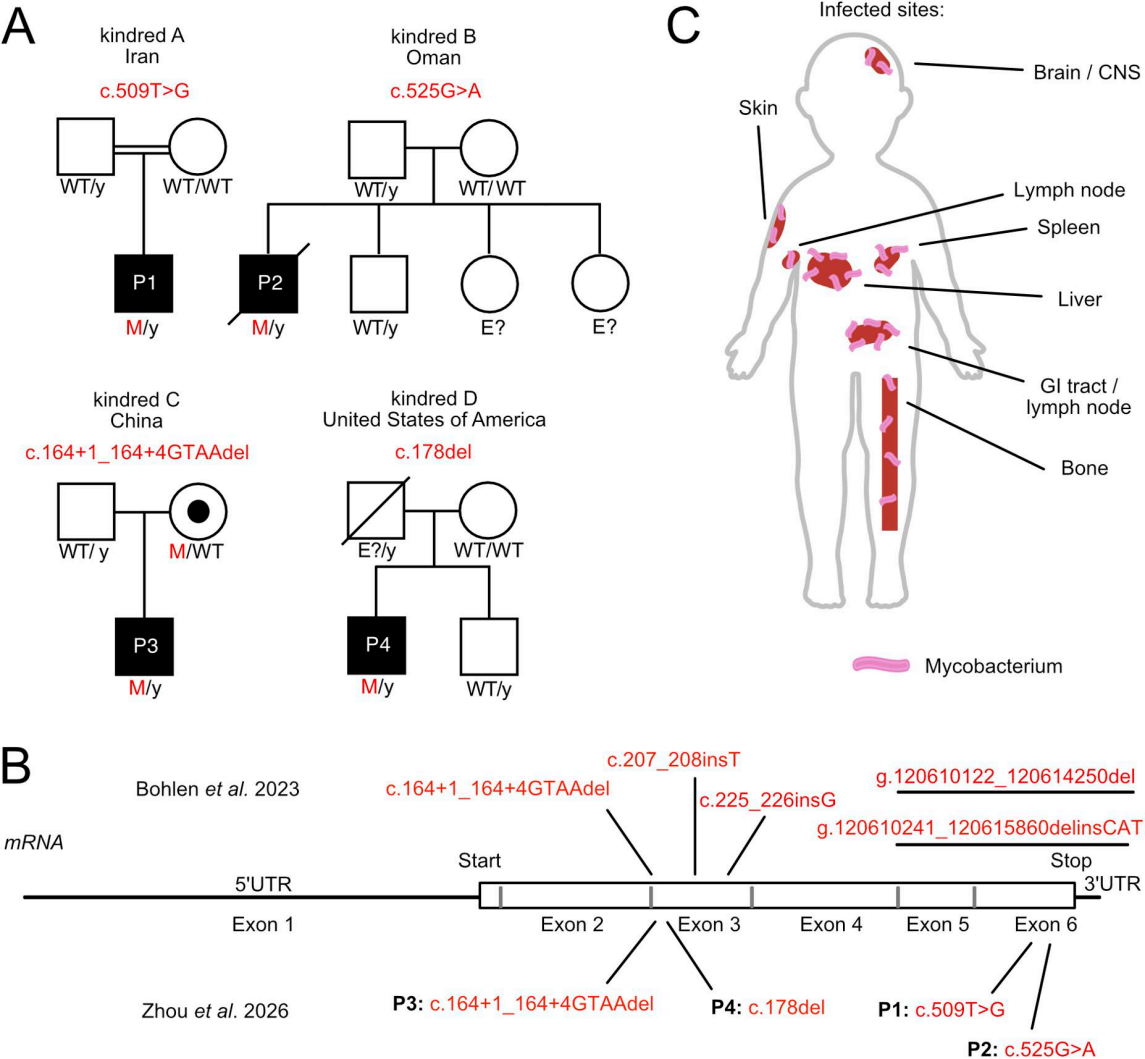
**New families with MSMD and MCTS1 variants. (A)** Family pedigrees with allele segregation for the four MSMD patients (black) hemizygous for the *MCTS1* variants c.509T>G, c.525G>A c.164+1_164+4GTAAdel, and c.178del, who suffered from BCG-osis (P1–P3) or *M. abscessus* infectious disease (P4). WT: wild-type, E?, unknown genotype; M, mutated; y, Y chromosome. **(B)** Summary of all the variants of *MCTS1* reported to date. **(C)** Diagram of organ/system infections observed in MCTS1-deficient patients in this report and in a previous study (8). CNS: central nervous system, GI: gastrointestinal.

Patient 1 (P1) was born in 2020 and is the only child of second-cousin Iranian parents (Fig. 1 A). BCG vaccination was administered on the day of birth. He was born prematurely at week 30, with a birth weight of 1,390 *g*. He had no family history of hereditary disease and was vaccinated according to the Iranian national program, which includes the BCG and oral polio vaccines. At the age of 4 mo, he presented BCG-osis with left axillary adenitis. Chest x-ray revealed an extrapulmonary soft-tissue mass in the left armpit. PCR for *M. bovis* complex yielded positive results for the axillary adenitis specimen and a bone marrow specimen from the right posterior iliac crest, as part of a systemic evaluation to rule out disseminated disease. An aspirated bone marrow sample was normocellular, with a myeloid:erythroid ratio of 3:1. P1 was treated with isoniazid and rifampicin, initially for 4 mo, but with subsequent prolongation for 18 mo based on his clinical response and microbiological status. Immunological analyses revealed that P1 had low immunoglobulin (IG) levels, despite having normal total serum protein and albumin levels. Intravenous IG (IVIG) treatment was therefore initiated. The lymphocyte transformation test (9, 10) revealed poor responses to BCG and *Candida* antigens but normal results with phytohemagglutinin (Table S4). At 3.5 years old, his weight was 10.5 kg, and he was 77 cm tall (both these growth parameters are below the third percentile). Currently, he remains well on ongoing IVIG and antimicrobial prophylaxis. He is on prophylaxis with azithromycin daily and IVIG monthly.

Patient 2 (P2) was born to consanguineous parents from Oman in 2016 (Fig. 1 A). He was vaccinated with BCG on the day of his birth. 7 mo later, he was diagnosed with abscessed BCG adenitis (BCG-itis). The abscess was drained, and the necrotic tissue was removed. P2 failed to thrive and had a height and weight below the third percentile. His gross motor development was delayed. At the age of 6 years, he presented chronic diarrhea, abdominal distension, and moderate ascites, but without fever. He was then hospitalized for a generalized tonic-clonic seizure. Cranial computed tomography (CT) and magnetic resonance imaging revealed otomastoiditis, acute cerebral venous thrombosis, and granulomatous meningitis with focal white matter lesions that were particularly abundant in the right hemisphere. The values of inflammatory parameters were high (C-reactive protein: 70 mg/L, white blood cell count: 24 × 10^9^/L, and neutrophils: 21 × 10^9^/L). Lymphopenia (lymphocytes: 1.07 × 10^9^/L) and anemia (hemoglobin: 8.8 g/dl) were observed. IG levels were normal, but IVIG was administered due to the patient’s severe medical condition, moderately low B cell counts and IgG levels (Tables S3 and S4). Despite intravenous phenytoin and dexamethasone treatment and anti-infection treatment with ceftriaxone, vancomycin, and acyclovir, the patient remained febrile and lethargic. He developed breakthrough seizures and was intubated and transferred to the pediatric intensive care unit. Cerebrospinal fluid (CSF) culture revealed the presence of pyrazinamide-resistant *M. bovis–*BCG. An empirical diagnosis of BCG-osis (affecting the brain, mastoid sinuses, lung, liver, and gastrointestinal tract) was established, and treatment with isoniazid, rifampicin, and ethambutol was initiated 11 days after admission. Echocardiography revealed heart failure, with a dilated left ventricle and moderate mitral valve regurgitation. *Aspergillus fumigatus* was also detected on CSF culture, leading to treatment with liposomal amphotericin, caspofungin. This regimen was transitioned to anidulafungin because caspofungin was unavailable. Total parenteral nutrition was administered due to continuous weight loss and pancolitis with diffuse edema and occasional ulcers. Neurological deficits, including spasticity, necessitated extensive limb physiotherapy. 2 mo later, the patient developed a status epilepticus. A cranial CT scan revealed a reduction of the edema in the right hemisphere and new lesions in the right frontal and temporal lobe, with acute or chronic meningitis. Subcutaneous IFN-γ1b treatment was initiated, leading to improvements in the patient’s activity, mobility, and nutritional status. However, the patient was subsequently lost to follow-up, leading to a cessation of IFN-γ1b treatment after 6 wk. He lost appetite and abdominal distension, hepatomegaly, and jaundice occurred. 3 mo later, he became lethargic for 1 wk without seizures. He developed fever and hematemesis, leading to his collapse and death within 24 h at the age of 6 years and 9 mo.

The third patient (P3) was born in China in 2012 and was vaccinated with BCG at birth. 1 mo later, he developed BCG-osis in the left armpit and received a traditional Chinese topical anti-infective therapy for about 3 mo. The local infection gradually improved, but P3 has suffered from recurrent diarrhea since the age of 6 years and recurrent otitis media since the age of 9 years. This recurrent diarrhea and abdominal pain led to a colonoscopy being performed at the age of 10 years, which revealed the presence of an ulcer in the ileocecum. A mucosal biopsy specimen from this area tested positive for *Mycobacterium tuberculosis* complex by metagenomic next-generation sequencing. Histopathology revealed small intestinal villous widening with epithelial damage and increased intraepithelial lymphocytes, focal crypt distortion in the colon, and mild chronic gastritis without granulomas; endoscopy showed a raised gastric lesion of unclear nature, negative for *Helicobacter pylori*, with ulcers in the small intestine and ileocecal valve, and signs of colitis. The patient subsequently received antimycobacterial treatment (isoniazid, rifampicin, ethambutol, and pyrazinamide), leading to an improvement in his condition. The patient is currently 13 years old and is well on antituberculosis treatment. He also receives IFN-γ-1b and IVIG due to his hypogammaglobulinemia (Tables S3 and S4).

The fourth patient (P4) was born in the United States of America (USA) in 2000. He is of Hispanic ancestry and was not vaccinated with BCG at birth. He had a healthy childhood, without frequent or notable infections. At the age of 16 years, he began to experience weight loss and diarrhea. At the age of 22 years, he presented intra-abdominal lymphadenopathy and underwent a laparoscopic excisional lymph node biopsy, which demonstrated the presence of microbial abscesses. Cultures of a subsequent laparoscopic excisional lymph node biopsy specimen led to the detection of *Mycobacterium abscessus* subspecies *abscessus*. The patient was diagnosed with disseminated *M. abscessus* infection manifesting as bulky intra-abdominal/retroperitoneal lymphadenopathy, chronic diarrhea, and chronic weight loss. The QuantiFERON test yielded negative results. Complete blood counts and clinical immunophenotyping results were normal (Tables S3 and S4). The patient has extensive mesenteric and retroperitoneal lymphadenopathy. The largest conglomerate within the retroperitoneum measures ∼8.3 × 5.0 × 7.3 cm and encases the abdominal aorta together with the proximal celiac and superior mesenteric arteries. There is also a more hypoattenuating component suggestive of intralesional necrosis. This patient also has a mild periportal edema in the liver and nonspecific calcifications in the spleen. He is currently 25 years old and on treatment with multiple antibiotics (imipenem + ceftaroline, omadacycline, clofazimine, and linezolid). Treatment with increasing doses of IFN-γ-1b for 1.5 years has started to show substantial improvements in the patient’s condition after ~1 year of treatment at a dosage of 400 µg three times per week.

### Private and predicted LOF variants of the *MCTS1* gene in the four patients

Using whole-exome sequencing (WES), we searched for non-synonymous, essential splice-site, or copy-number variants (CNV) of the *MCTS1* gene*.* In this manner, we identified hemizygosity for two new nonsense variants, one new frameshift variant and one known small-deletion variant (NM_014060.3, GRCh38 chr X) in these four patients with MSMD. The variant of P1, c.509T>G, is located in exon 6 and predicted to be p.L170* (p.Leu170*). The variant of P2, c.525G>A, is located in exon 6 and predicted to be p.W175* (p.Trp175*). The p.L170* and p.W175* variants affect only the extreme C terminus of the protein. The variant of P3 is c.164 + 1_164+4GTAAdel (predicted to encode p.K4_C55delinsN), which we previously found in a Saudi Arabian family and showed to be LOF due to defective splicing of the *MCTS1* mRNA (8). The occurrence of this allele in two unrelated families (from China and Saudi Arabia) suggests that this is a mutation hotspot. Indeed no haplotype common to these two patients was found in the region of this variant (Table S5). The variant of P4, c.178del, is located in exon 3 and predicted to be p.E60Kfs5* (p.Glu60Lysfs*5) (Fig. 1 B). Tests on the parents and close relatives of these patients yielded the expected familial segregation for the *MCTS1* variants, confirming XR inheritance (Fig. 1 A). However, the *MCTS1* variants were not found in DNA from the whole blood of the mothers of P1 and P2, suggesting the variants occurred de novo in these kindreds. In the updated gnomAD V4.1 (>807,000 WES/WGS), there are four female, heterozygous carriers of predicted LOF (pLOF) variants of MCTS1. There is one male, hemizygous carrier of a pLOF variant that affects an alternative splice form of MCTS1. This variant is not predicted to affect the canonical transcript and protein. None of the pLOF variants reported in this manuscript are found in the gnomAD database, and they are therefore considered private. Potential other candidate variants for patients P1, P2, and P3 are listed in Tables S6, S7, and S8. Variants for P4 were acquired and analyzed in a clinical diagnostic laboratory in the USA, and raw data are not available to us. For P4, no other variants in genes related to his phenotype were identified, and neither were any American College of Medical Genetics and Genomics secondary findings. Our findings indicate that patients with rare, hemizygous pLOF *MCTS1* variants are susceptible to mycobacterial disease (Fig. 1 C).

### The variants of two of the patients lead to the production of a truncated MCTS1 protein

**Figure 2.**
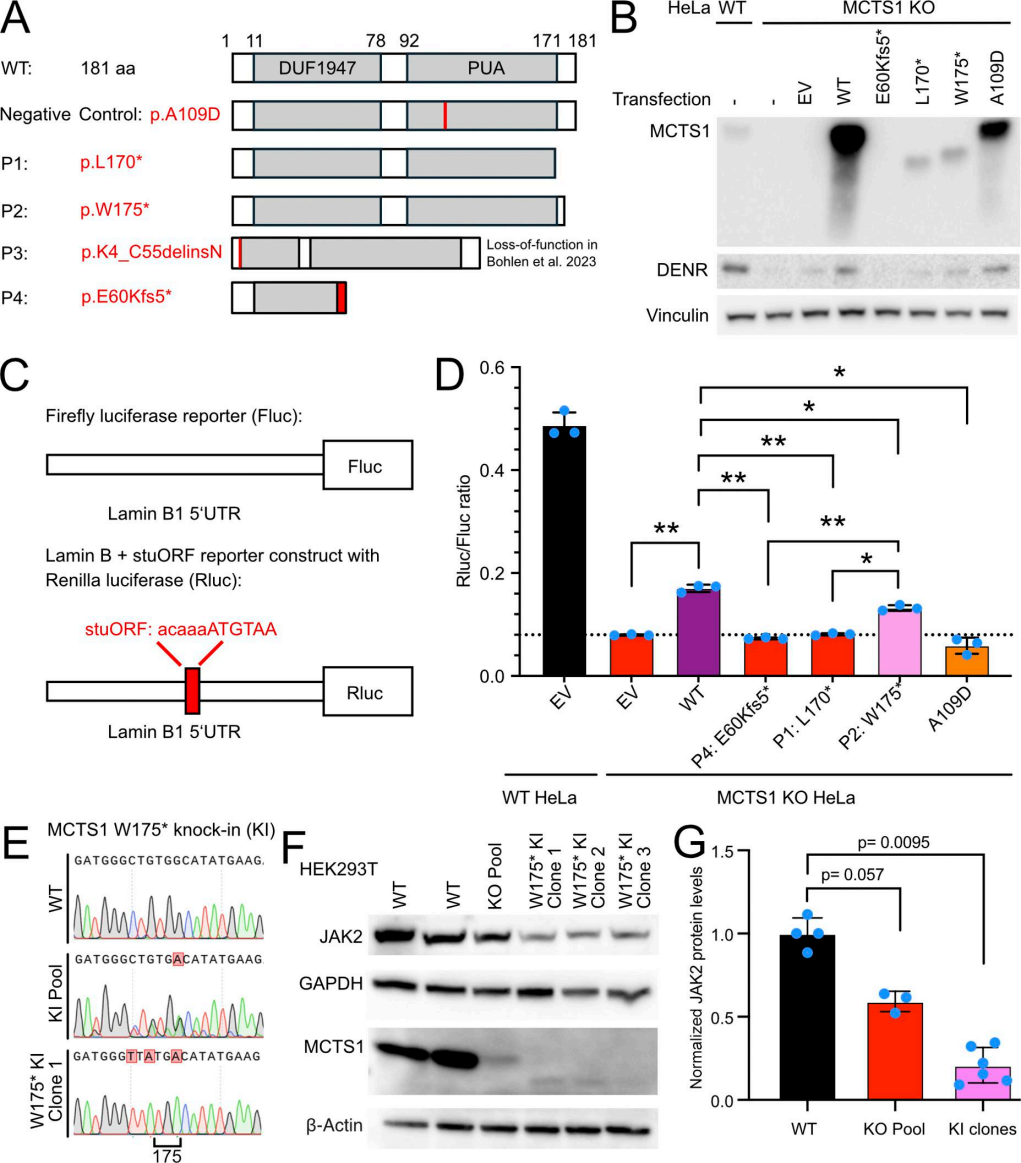
**Functional investigation of the new MCTS1 variants. (A)** Predicted structure of the MCTS1 protein with the domain of unknown function 1947 (DUF1947) and the pseudouridine synthase and archaeosine transglycosylase (PUA) domains for WT protein, the synthetic LOF variant p. A109D, and the patient variants p.L170* (P1), p.W175* (P2), and predicted p.E60Kfs5* (P4). The numbers annotated for the WT MCTS1 protein correspond to the first and last amino acids of the MCTS1 protein, the DUF1947, and PUA domains. The variants of MCTS1 are shown in red. **(B)** Western blot of WT and MCTS1-KO HeLa cells after transfection with EV, WT MCTS1 (pWT), or the MCTS1 variants p.E60Kfs5* (pE60Kfs5*), p.L170* (pL170*), p.W175* (pW175*), and p.A109D (pA109D). Details are provided in Table S2. Non-transfected HeLa cells (“−”) were used as a negative control. **(C)** Schematic diagram of the Fluc and lamin B + stuORF reporters used to assess MCTS1 activity. stuORF, synthetic strong Kozak uORF. Modified from (8). **(D)** Activity of the MCTS1 variants in the luciferase translation reinitiation assay after the transfection of WT and MCTS1-KO cells with EV, WT MCTS1 or the various MCTS1 variants (p.E60Kfs5* p.L170*, W175*, and p.A109D; see details in Table S2). Bars: Mean and standard deviation of three technical replicates, represented as turquoise dots. This experiment is representative of the three biological replicates. The asterisks indicate the level of significance, as assessed in a one-way ANOVA with Tukey’s test for multiple comparisons and adjustment for multiple testing (*P < 0.05; **P < 0.005). **(E)** Sanger sequencing confirmation of KI of the MCTS1 W175* variant and silent L174L variants in HEK293T cells. **(F)** Western blot of HEK293T cells subjected to genome editing to introduce MCTS1 KO in a pooled manner or MCTS1 W175* KI with the indicated single-cell derived clones shown. **(G)** Quantitation of three independent western blots of the indicated cell lines. Statistical significance was assessed using two-sided Mann–Whitney tests. Source data are available for this figure: SourceData F2.

We then assessed the impact of the three new MCTS1 variants, p.E60Kfs5*, p.L170*, and p.W175*, on protein expression. We overexpressed these variants in MCTS1^KO^ HeLa cells. For p.L170* and p.W175*, we expected to detect MCTS1 proteins truncated at the C terminus by 12 and 7 amino acids, respectively (Fig. 2 A). We performed western blotting to determine the levels of these MCTS1 variants overexpressed in MCTS1^KO^ HeLa cells. The p.E60Kfs5* variant resulted in a complete LOE, whereas the nonsense variants resulted in substantially lower protein levels (Fig. 2 B). Consistent with expectations, the p.L170* variant was detected at a lower molecular weight than the p.W175* variant. Both were smaller than the wild-type (WT) MCTS1 protein (Fig. 2 B). Thus, two of the three new MCTS1 variants are not LOE but lead to the production of very small amounts of a truncated MCTS1 protein.

### MCTS1 p.E60Kfs5* and p.L170* are LOF, whereas p.W175* is hypomorphic for translation reinitiation

MCTS1 forms a complex with the DENR protein to remove the transfer RNA (tRNA) bound to the stop codon in the 40S ribosome. This is crucial for the reinitiation of translation. Some mRNAs have an upstream ORF (uORF) in the 5′ untranslated region (5′UTR) in addition to the main protein-coding ORF (mORF) (11). The MCTS1–DENR complex is therefore required to remove the tRNA from the stop codon of the uORF before the translation of the mORF can occur (11, 12, 13). We hypothesized that these new MCTS1 variants might be LOF for translation reinitiation. We tested the functional consequence of the MCTS1 variants of P1 and P2 in a luciferase-based translation reinitiation assay (Fig. 2 C) (14). In MCTS1^KO^ HeLa cells, the p.E60Kfs5* and p.L170* variants had activity levels similar to those of the two negative controls, empty vector (EV) and A109D, suggesting that these variants are LOF. However, a different situation was observed for the p.W175* variant of P2, which yielded a higher normalized renilla luciferase (Rluc)/firefly luciferase (Fluc) ratio than the LOF variants p.L170* (P = 0.012), A109D (P = 0.021), and the EV (P <0.001), but a lower normalized Rluc/Fluc ratio than that observed after transfection with WT MCTS1 (P = 0.031) (Fig. 2 C). Thus, the p.W175* variant is hypomorphic relative to the variants previously described in patients (8) and the new LOF variants p.E60Kfs5* and L170*. The complete or partial deficiency of MCTS1 activity of the variants in the luciferase assay may account for the severe mycobacterial diseases of the Iranian (P1), Omani (P2), Chinese (P3), and American (P4) patients.

### Substantially reduced JAK2 levels in HEK293T clones with MCTS1 W175* knock-in (KI)

We aimed to investigate the functional consequence of the hypomorphic MCTS1 variant W175* on JAK2 protein levels. To this end, we used CRISPR/Cas9 and homology-driven repair (HDR) to introduce this genotype in homozygosity at the endogenous *MCTS1* locus of HEK293T cells (Fig. 2 E). We introduced a silent L174L variant in front of the patient’s variant to avoid repeated genomic DNA (gDNA) cutting by the Cas9 enzyme. Western blotting of these cells in comparison to a MCTS1 knockout (KO) pool (8) that has 10–20% residual MCTS1 expression reveals a strong depletion of full-length MCTS1 and the presence of a very weak band showing a truncated protein (Fig. 2 F). This band is consistent with the observations from MCTS1 overexpression (Fig. 2 B). Finally, quantification of JAK2 protein levels confirmed that the W175* genotype potently reduced JAK2 expression levels (Fig. 2, F and G), suggesting that it may behave essentially equivalent to a complete deficiency when present in the endogenous *MCTS1* locus.

## Discussion

We report here four new patients from four unrelated families with XR MCTS1 deficiency originating from and living in China, Iran, Oman, and the USA. Two of the new variants resulted in the production of residual amounts of a truncated MCTS1 protein in an overexpression system. One *MCTS1* allele, p.W175* was hypomorphic, suggesting that a decrease in MCTS1 activity, as opposed to its total abolition, may be sufficient to cause susceptibility to mycobacterial disease. The residual activity of the overproduced p.W175* MCTS1 protein may result in a de facto complete LOF for endogenous expression in the cells of the patient, as indicated by our results from KI cells. Unfortunately, we were unable to evaluate the endogenous effects of this variant in the patient due to his death. We also report the occurrence of a previously described essential splice-site variant in intron 2 of the gene in a Chinese family. This variant was previously detected in a Saudi Arabian family, and its occurrence in two different populations suggests the existence of a mutation hotspot, as opposed to a founder effect.

As in our original cohort, we observed substantial heterogeneity in the severity of BCG disease (8). Three of the patients reported here had BCG-osis during infancy after BCG vaccination, whereas the final patient was not vaccinated with BCG and developed EM infection by *M. abscessus* during adolescence. Additionally, a central nervous system (CNS) infection with *A. fumigatus* was observed in P2. CNS aspergillosis is potentially a new infectious phenotype of MCST1 deficiency, although with only one case, this remains to be validated. Out of 876 MSMD patients reviewed in the literature, only three patients developed aspergillosis. This infectious disease is thus rare but present in MSMD patients. *Aspergillus* is an intracellular fungus living on the macrophages (15, 16). Thus, the activity of IFN-γ is likely important for the restriction of *Aspergillus*. The clinical expression of BCG infectious disease was markedly heterogeneous, ranging from regional lymphadenitis to disseminated disease. This clinical heterogeneity may result partly from the residual expression and function of MCTS1, but may also be due to modulating genetic or environmental factors that should be investigated in future studies. In particular, chronic diarrhea was documented in three of the four patients. The extensive mesenteric and retroperitoneal lymphadenopathy of the P4 may have contributed to the development of protein-losing enteropathy. Similar conditions have been described in IL-12Rβ1–deficient patients with several enlarged lymph nodes in the abdomen (17, 18). Additional studies are required to determine the underlying mechanism.

So far, we have identified eight different genotypes from six countries suffering from MSMD due to an MCTS1 deficiency. In seven cases, the disease was caused by BCG. However, the patient from the USA in this study had an infectious disease due to an EM, providing insight into the likely clinical and infectious trajectories of individuals with MCTS1 deficiency in whom susceptibility is not triggered by BCG vaccination. Remarkably, this patient grew up completely healthy until the age of 16 years. A negative QuantiFERON test result indicated that this patient has not yet been exposed to *M. tuberculosis*, consistent with his residence in a country in which this *Mycobacterium* is not endemic. These findings highlight the importance of detecting MCTS1 variants in clinical sequencing panels for the identification of inborn errors of translation reinitiation, particularly in patients with isolated mycobacterial disease, whether due to BCG or EM. We have now identified nine families from six countries, as diverse as China, Finland, Iran, Oman, Saudi Arabia, and the USA, with eight genotypes. Any candidate MCTS1 variants detected should be subjected to functional evaluations, as even hypomorphic variants can cause clinical disease. MCTS1-deficient patients with acute mycobacterial infections should receive multiple antibiotics against mycobacteria and subcutaneous recombinant IFN-γ-b1, as they have defects in the production of this cytokine. Vaccination with live BCG is contraindicated when MCTS1 deficiency is suspected or confirmed.

## Materials and methods

### Human patients

Informed consent for the processing of patient-related data and samples was obtained in the countries of residence of the patients (Iran for kindred A [P1, Tehran University of Medical Sciences Ethics Committee, IR.TUMS.CHMC.REC.1398.030], Oman for kindred B [P2, Health Studies and Research Approval Committee Oman, MoH/CSR/25/31701], China for kindred C [P3, Ethics Committee of the Children’s Hospital of Fudan University, No.[2022] 100], and USA for kindred D [P4, University of California San Francisco Institutional Review Board, Study number 18-25891]) in accordance with local regulations and with the approval of the institutional review board. The physicians caring for the patients and their families provided information about the patients’ clinical features, including diagnostic procedures, laboratory findings, and treatment, on a pseudonymized form. Primary cells of the four patients were not available for the present study.

### Cell lines and culture

WT HeLa cells (Cat# CRM-CCL-2; ATCC, RRID: CVCL_0030) and MCTS1 KO HeLa cells (11) were cultured in Dulbecco/Vogt modified Eagle’s minimal essential medium (DMEM, Gibco) with 10% fetal-calf serum (FCS) and 100 IU/ml penicillin/streptomycin (Cat# 15140122; Gibco). We used 0.25% trypsin-EDTA (Cat# 25200-56; Gibco) to detach the cells for subculture.

### Primers and plasmids

All the primers and plasmids used in this study are listed in Tables S1 and S2. The pL170*, pW175*, and pE60Kfs5* plasmids, containing the MCTS1 variants from the MSMD patients, were generated by PCR mutagenesis. We used the pRK plasmid with the WT *MCTS1* cDNA inserted into the gene expression cassette (8) as the DNA template. We used OJB365 and OJB366 as primers for the p.L170* variant, OJB367 and OJB368 for p.W175*, and OJB397 and OJB398 for p.E60Kfs5* (Table S1). PCR was performed with the “CloneAmp HiFi PCR Premix” (Cat# 639298; CloneTech). PCR products of about 5 kb in size were purified by electrophoresis in 1% agarose gels, followed by isolation of the corresponding bands with the NucleoSpin Gel and PCR Clean-up Kit (REF: 740609; Macherey-Nagel). The linear PCR products were then inserted into plasmids with the InFusion HD Cloning Kit (Cat# 102518; Takara). The new plasmids were used to transform NEB Stable Competent *E. coli* (High Efficiency) (Cat# C3040H; New England Biolabs) and were then extracted with the QIAprep Spin Miniprep Kit (Cat#27104; QIAGEN). The presence of the studied variants was confirmed by Sanger sequencing with two different primers, OTC33 and OJB273 (Table S1), and the Big Dye Terminator v3.1 cycle sequencing kit (Cat# 4337455; Applied Biosystems) on an ABI Prism 3700 sequencer (Applied Biosystems). All procedures were performed in accordance with the manufacturers’ instructions.

### Translation reporter assay

We used WT and KO MCTS1 HeLa cells to seed 96-well plates at a density of 15,000 cells per well. The cells in each well were transfected with 5 μl of a transfection mixture containing 0.3 μl X-tremeGENE 9 Transfection Reagent (Cat# 6365779001; Roche), 6.25 ng firefly luciferase plasmid (Table S2), 6.25 ng of one of the *Renilla* luciferase plasmids (either pLamin B 5′UTR or pLamin B 5′ UTR + stuORF, Table S2), and 37.5 ng of one of the test plasmids (pGFP, pEV, pWT, pE60Kfs5*, pL170*, pW175*, and pA109D, Table S2). We added OptiMEM I Reduced Serum Medium (Cat# 31985070; Gibco) to the transfection mixture to obtain a final volume of 5 μl per well. The transfection mixture was incubated for 45 min before being added to the cells. Firefly and *Renilla* luciferase activities were determined 16–20 h after transfection with the Dual-Luciferase Reporter Assay System (Cat# E1910; Promega) and a VICTOR-X Multilabel Plate Reader (PerkinElmer Life Sciences) used in accordance with the manufacturer’s instructions. Transfection was performed with technical triplicates, and three biological replicates were performed.

### Production of W175* KI cells

HEK293T cells endogenously expressing MCTS1 W175*were generated using the CRISPR-Cas9 KI technology. First, small guide RNA (sgRNA) (5′-AUU​AUU​UAA​AUG​AUG​GGC​UG-3′) and Cas9 enzyme were mixed in equal amounts (7.5 pmol) and incubated at room temperature for 10 min 15 pmol of single stranded DNAtemplate (ssDNAtemplate) (5′-GAA​TTG​GCA​TTG​AAA​ATA​TCC​ATT​ATT​TAA​ATG​ATG​GGT​TAT​GAC​ATA​TGA​AGA​CAT​ATA​AAT​GAG​CCT​CAG​AAG​GAA​TGC-3′) was subsequently added to the mix. Meanwhile, 500,000 cells per reaction were washed once with PBS and resuspended in Editing buffer from the Nucleofactor Solution Set SF kit (#PBC2-02250; Lonza). Ribonucleoprotein complex with ssDNAtemplate was added to the cells, and the whole mixture was pipetted into the Lonza electroporation chamber. Cells were electroporated with the Lonza 4D-Nucleofactor Core Unit (Pulse Code: CM 130) and immediately resuspended in DMEM media supplemented with 10% FCS, 1% (100 IU/ml) of penicillin/streptomycin, and 1 µM Alt-R HDR Enhancer V2 (Integrated DNA technologies). Editing efficiency was determined with Sanger sequencing 24 h after electroporation, and cells were resuspended fresh DMEM media. Cells were diluted to 1 cell per 100 μl and seeded in multiple 96-well Flat-bottom plates. Single clones were grown and Sanger sequenced to confirm homozygous KI of the patient variant.

### Immunoblotting

We used WT and KO MCTS1 HeLa cells to seed 6-well plates at a density of 225,000 cells per well. For transfection, 100 μl transfection mixture, consisting of 1 μ g plasmid DNA (pGFP, pEV, pWT, pE60Kfs5*, pL170*, pW175*, or pA109D, Table S2) and 3 μl X-tremeGENE 9 Transfection Reagent in OptiMEM I Reduced Serum Medium, was added to the 2 ml of cell culture per well. We washed the cells three times with PBS 16–20 h after transfection and lysed them with Pierce radioimmunoprecipitation assay (RIPA) buffer (Cat# 89901; Thermo Fisher Scientific) supplemented with Complete Mini, EDTA-free protease inhibitor cocktail (Cat# 11836170001; Roche, 1 tablet in 10 ml), PhosStop phosphatase inhibitor cocktail (Roche; Cat# 4906837001, 1 tablet in 10 ml), 100 mM Thermo Fisher Scientific PMSF Protease Inhibitor (Cat# 36978; Thermo Fisher Scientific), and 0.025 M β-glycerophosphate (Sigma-Aldrich) together with 1 μl Benzonase nuclease (50,000 IU/ml, Cat# E1014; Sigma-Aldrich). The protein concentration of the cell lysates was determined with the Pierce protein assay kit (Cat# 23225; Thermo Fisher Scientific) and a VICTOR-X Multilabel plate reader, according to the manufacturers’ instructions. For sodium dodecyl sulfate-polyacrylamide gel electrophoresis on 12% Criterion TGX Precast Gels (Cat# 5671045; Bio-Rad), the cell lysates were diluted to equal protein concentrations with Pierce RIPA buffer and 4× Laemmli Sample Buffer (Cat# 161-0747; Bio-Rad). Following electrophoresis, the protein bands were electroblotted onto a nitrocellulose membrane with 0.2 m pores (Trans-Blot Turbo Midi 0.2 μm Nitrocellulose Transfer Pack, Cat# 1704159; Bio-Rad) at 1 A and 25 V for 30 min and subjected to Ponceau staining. The nitrocellulose membranes were blocked by incubation with 5% skim milk in 0.1% Tween 20 in PBS (PBST) for 1 h at room temperature before overnight incubation with the primary antibodies at 4°C. The primary antibodies used were guinea pig anti-human MCTS1 and guinea pig anti-human DENR antibodies (both kindly provided by the Teleman laboratory) and rabbit anti-human JAK2 antibody (Cat# 3230; Cell Signaling Technology) or Beta Actin antibody (C4, HRP:sc-47778) at a dilution of 1:1,000 in 5% skim milk in PBST. The membrane was then washed three times, for 15 min each, with PBST before incubation with the secondary antibodies for 1 h at room temperature. The secondary antibodies used were horseradish peroxidase (HRP)–coupled goat anti-guinea pig (Cat# A18769; Invitrogen) and stabilized peroxidase-conjugated goat anti-rabbit antibodies (Cat# 32460; Invitrogen), each at a dilution of 1:5,000 in 5% skim milk in PBST. The nitrocellulose membranes were then washed again three times, for 15 min each, with PBST. Chemiluminescence was detected with the Clarity western ECL substrate (Cat# 1705061; Bio-Rad) and the ChemiDoc imaging system (Cat# 12003153; Bio-Rad). The membranes were stripped by incubation for 20 min with Restore western blot stripping buffer (Cat# 21063; Thermo Fisher Scientific) and stained for glyceraldehyde 3-phosphate dehydrogenase (GAPDH) and vinculin as described for MCTS1 and JAK2, but with the mouse anti-human GAPDH (Cat# sc-47724; Santa Cruz) and HRP-coupled mouse anti-vinculin (7F9) (Cat# sc-73614 HRP; Santa Cruz) antibodies at a 1:5,000 dilution as the primary antibodies and HRP-conjugated goat anti-mouse IgG (Cat# 1706516; Bio-Rad) at a dilution of 1:5,000 as the secondary antibody. The nitrocellulose membranes were incubated with the primary antibodies for 1 h at room temperature. Two replicates were performed.

### Statistics

Statistical analyses were performed with R Studio (Version 2023.06.0 + 421, Posit Software) and IBM SPSS Statistics (Version 29.0.2.0, IBM). Shapiro–Wilk tests were used to evaluate the normality of the data distribution. The translation reporter assay data were analyzed by one-way analysis of variance with Dunnett T3 tests for multiple comparisons. The error bars indicate the SD. We considered P values <0.05 to be statistically significant. Significance is indicated as follows: * for P < 0.05, ** for P < 0.01, and *** for P < 0.001.

### Online supplemental material

We provide the following supplemental materials for this manuscript: Table S1 shows the sequences of the primers used in this study. Table S2 shows the plasmids used in this study. Table S3 shows the hematological assessment of the patients. Table S4 shows the immunological assessment of the patients. Table S5 shows the overlap in variants between P1 and P3. Table S6 shows the homozygous variants and heterozygous pLOF variants present in P1. Table S7 shows the homozygous non-synonymous and heterozygous pLOF variants present in P2. Table S8 shows the additional candidate variants present in P3.

## Supplementary Material

SourceData F2 is the source file for Fig. 2.

Table S1 shows the sequences of the primers used in this study.

Table S2 shows the plasmids used in this study.

Table S3 shows the hematological assessment of the patients.

Table S4 shows the immunological assessment of the patients.

Table S5 shows the overlap in variants between P1 and P3.

Table S6 shows the homozygous variants and heterozygous pLOF variants present in P1.

Table S7 shows the homozygous non-synonymous and heterozygous pLOF variants present in P2.

Table S8 shows the additional candidate variants present in P3.

## Data Availability

All the data will be made available, upon request, by the corresponding authors.
