# Supplementary figures and images for: Complete and partial forms of X-linked MCTS1 deficiency in patients with mycobacterial disease
Source: J Hum Immun. 2026 Jan 30;2(2):e20250073. doi: 10.70962/jhi.20250073 (PMC12857535; doi:10.70962/jhi.20250073)

Figure 2B

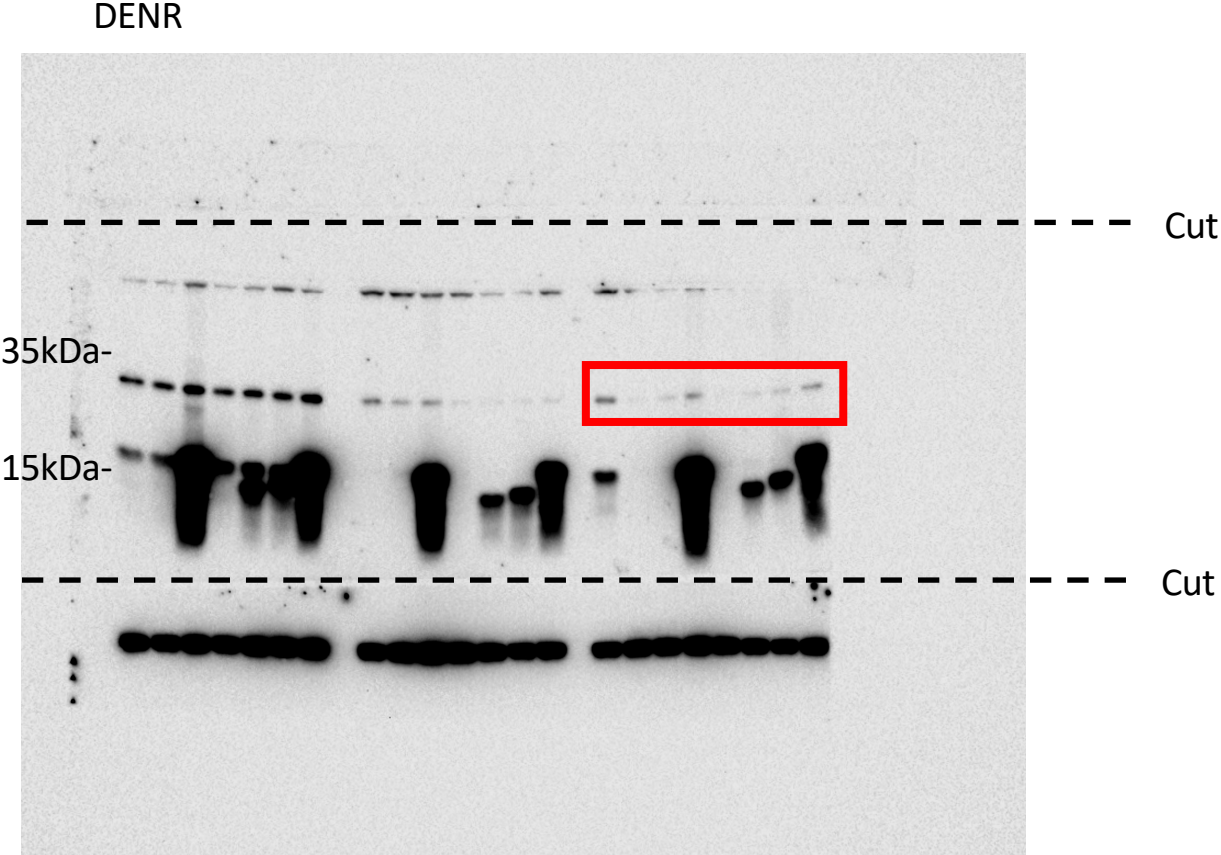

Figure 2B

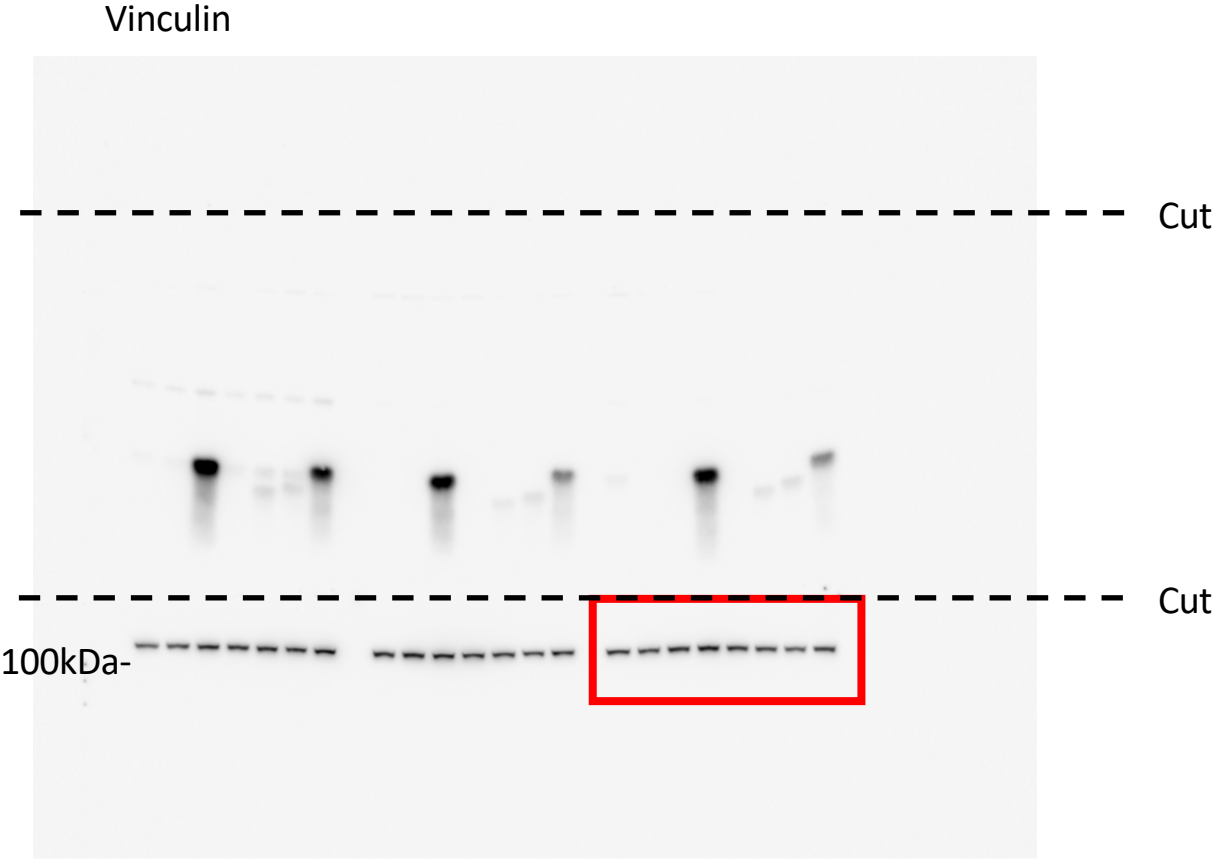

Figure 2B

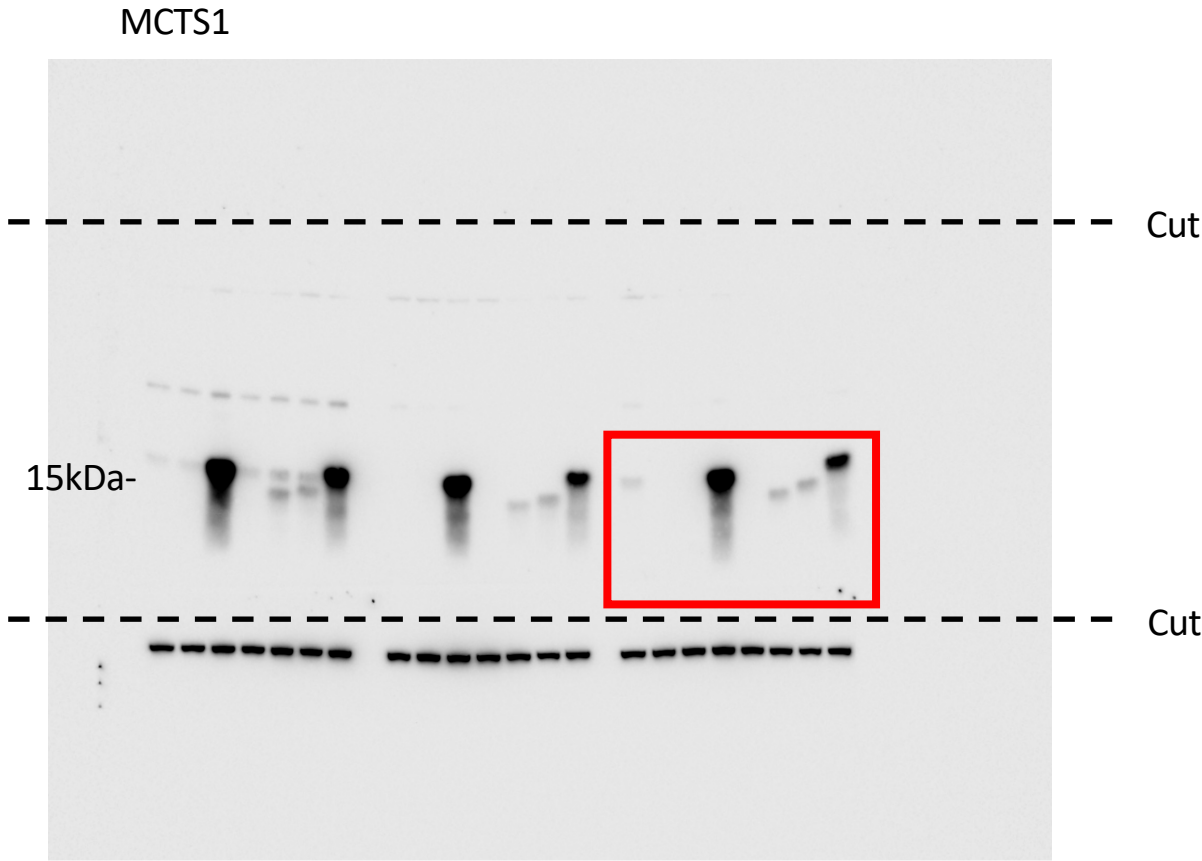

Figure 2F

Actin

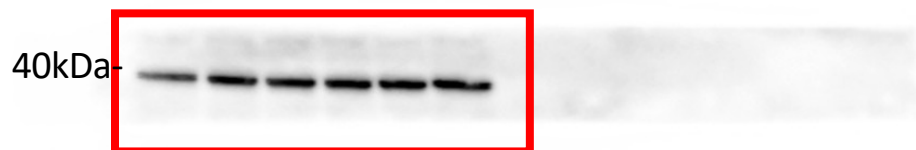

Figure 2F

MCTS1

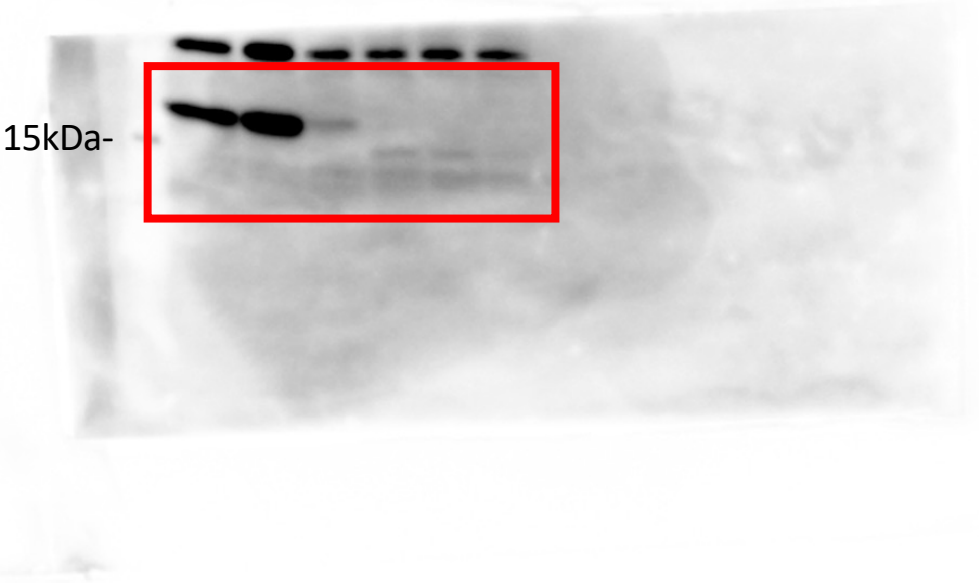

Figure 2F

JAK2

100kDa-

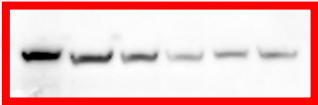

Figure 2F

GAPDH

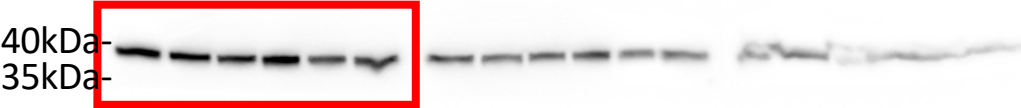

Supplement: SourceData F2 — is the source file for Fig. 2. [file jhi_20250073_sourcedataf2.pdf]
