# Supplementary material for: Complete and partial forms of X-linked MCTS1 deficiency in patients with mycobacterial disease
Source: J Hum Immun. 2026 Jan 30;2(2):e20250073. doi: 10.70962/jhi.20250073 (PMC12857535; doi:10.70962/jhi.20250073)
Supplement: Table S1 — shows the sequences of the primers used in this study. [file jhi_20250073_tables1.docx]

**Table S1:** Sequences of the primers used in this study

| **Primer** | **Sequence (5’ 🡪 3’)** |
| --- | --- |
| OJB273 | ATGGATATCTCAGAATCATTTATATTCTTCATG |
| OJB365 | ATCCATTATTGAAATGATGGGCTGT |
| OJB366 | CCATCATTTCAATAATGGATATTCT |
| OJB367 | GATGGGCTGTGACATATGAAGA |
| OJB368 | TCATATGTCACAGCCCATCATT |
| OTC33 | AAATGGGCGGTAGGCGTG |
| OJB397 | CATGAACATATAAAATCCTTACAGATG |
| OJB398 | ACTGTAAGGATTTTATATGTTCATGGCATC |
