# Supplementary material for: Complete and partial forms of X-linked MCTS1 deficiency in patients with mycobacterial disease
Source: J Hum Immun. 2026 Jan 30;2(2):e20250073. doi: 10.70962/jhi.20250073 (PMC12857535; doi:10.70962/jhi.20250073)
Supplement: Table S2 — shows the plasmids used in this study. [file jhi_20250073_tables2.docx]

**Table S2:** Plasmids used in this study

| **Plasmid** | **Description** |
| --- | --- |
| pEV | pRK backbone alone, with no insertion [^8^](#_ENREF_8) |
| pWT | backbone: pRK, insertion: wildtype MCTS1 cDNA in the gene expression cassette [^8^](#_ENREF_8) |
| pE60KfsX5 | backbone: pRK, insertion: MCTS1 cDNA with the frameshift variant p.Glu60Lysfs*5 (c.178del) in the gene expression cassette |
| pL170X | backbone: pRK, insertion: MCTS1 cDNA with the nonsense variant p.Leu170* (c.509T>G) in the gene expression cassette |
| pW175X | backbone: pRK, insertion: MCTS1 cDNA with the nonsense variant p.Trp175* (c.525G>A) in the gene expression cassette |
| pA109D | backbone: pRK, insertion: MCTS1 cDNA with the missense variant p.Ala109Asp [^10^](#_ENREF_10) |
| pGFP | pcDNA^TM^3.1 ^(+)^ Mammalian Expression Vector (Invitrogen, Cat# V79020) with the GFP coding sequence |
| pLamin B 5’UTR | pRL-CMV vector (Promega, Cat# E226A) with the SpeI restriction site removed and the 5’UTR (5’ untranslated region) of human lamin B1 with addition SpeI and AgEI restriction sites inserted. [^12^](#_ENREF_12) |
| pLamin B 5’ UTR + stuORF | Insertion of the synthetic strong-Kozak upstream open reading frame (stuORF, sequence: acaaaATGTAA) into the human lamin B1 5’UTR of pLamin B 5’UTR. [^12^](#_ENREF_12) |
| pFluc | The pRL-CMV vector (Promega, Cat# E226A) was digested with NheI and XbaI to replace the RLuc ORF withthe Fluc ORF. The HindIII and NheI restriction sites were then used to insert thelLamin B1 5’UTR upstream the FlucORF. [^12^](#_ENREF_12) |
