# Supplementary material for: Complete and partial forms of X-linked MCTS1 deficiency in patients with mycobacterial disease
Source: J Hum Immun. 2026 Jan 30;2(2):e20250073. doi: 10.70962/jhi.20250073 (PMC12857535; doi:10.70962/jhi.20250073)
Supplement: Table S3 — shows the hematological assessment of the patients. [file jhi_20250073_tables3.docx]

**Table S3:** Haematological assessment of the patients

| **Evaluation 1** | **P1** | **P2** | **P3** | **P4** |
| --- | --- | --- | --- | --- |
| **Sex** | male | male | male | male |
| **Origin (country)** | Iran | Oman | China | USA |
| **Follow-up (alive or dead)** | alive, 3.5 y.o. | died at 6 years & 10 months | alive, 11 y.o. | alive, 24 y.o. |
| **Genetic testing- MCTS1 allele** | c.509T>G | c.525G>A | c.164+1_164+4GTAAdel | c.178Gdel |
| **Predicted MCTS1 protein** | p.L170* | p.W175* | p.K4_C55delinsN | p.E50Ffs*5 |
| **Loss-of expression (LOE)** | substantially reduced | substantially reduced | yes | yes |
| **Loss-of-function (LOF)** | yes | hypomorphic | yes | yes |
| **Hematological data** |  |  |  |  |
| **Age at evaluation (years)** | 4 months | 6 years | 11, acute infection | 24 years |
| **Neutrophils** (number and percentage) | 3,731 (21.2%) | 7,100 (81%) | 13110 (78%) | 1,600 (31%) |
| **Eosinophils** (number and percentage) | N.A. | N.A. | 270 | 200 (4%) |
| **Basophils** (number and percentage) | N.A. | N.A. | 60 | 100 (2%) |
| **Monocytes** (number and percentage) | 1,420 (12.1%) | 400 (4.5%) | 1350 (8%) | 500 (10%) |
| **Lymphocytes** (number and percentage) | 11,880 (67.5%) | 800 (9%) | 2030 (12%) | 2,600 (53%) |
| **Platelets** | 672 000 | 269 000 | 291 000 | 141 000 |
| **Hematocrit/Hemoglobulin** | N.D. (11.2) | 31.8% (10.1) | N.D. (14.4) | 33.5% (11.5) |
|  |  |  |  |  |
| **Evaluation 2** | **P1** | **P2** | **P3** | **P4** |
| **Age at evaluation (years)** | 3 years, stable | 6 years & 2 months | 12 years, stable |  |
| **Neutrophils** (number and percentage) | 2122 (18.3%) | 7700, 61% | 2420 (60%) |  |
| **Eosinophils** (number and percentage) | 56 (0.04%) | 100, 0.9% | 380 (9%) |  |
| **Basophils** (number and percentage) | 75 (0.06%) | 200, 1.7% | 70 (1.7%) |  |
| **Monocytes** (number and percentage) | 920 (7.9%) | 1100, 9.6% | 230 (5.7%) |  |
| **Lymphocytes** (number and percentage) | 9268 (79.9%) | 2400, 21% | 950 (23.5%) |  |
| **Platelets** | 383 000 | 747 000 | 216 000 |  |
| **Hematocrit/Hemoglobulin** | 34. 6% (12.1) | 31% (10.2) | N.D. (13.2) |  |
