# Supplementary material for: Complete and partial forms of X-linked MCTS1 deficiency in patients with mycobacterial disease
Source: J Hum Immun. 2026 Jan 30;2(2):e20250073. doi: 10.70962/jhi.20250073 (PMC12857535; doi:10.70962/jhi.20250073)
Supplement: Table S4 — shows the immunological assessment of the patients. [file jhi_20250073_tables4.docx]

**Table S4:** Immunological assessment of the patients

| Evaluation 1 | P1 | P2 | P3 | P4 |
| --- | --- | --- | --- | --- |
| Age at evaluation (years) | 4 months | 6 years | 11 years, acute infection | 23 years |
| T lymphocytes (number and percentage) | 7104, 59.7% | 510, 78% | 853, 56,58% | 2714; 82% |
| B lymphocytes (number and percentage) | 3142, 26.4% | 60, 9% | 60, 4% | 265; 8% |
| NK cells (number and percentage) | 1247, 10.5% | 20, 3% | 582, 38.5% | 298; 9% |
| IgG (g/L) | 11.6 | 3.7 | 1 | 18.9 |
| IgM (g/L) | 0.19 | 0.36 | 0.13 | 0.82 |
| IgA (g/L) | 0.09 | 0.94 | 0.15 | 2.42 |
| IgE (IU/mL) | 1.7 | 11 | 11.5 | n/a |
| DHR |  | Normal |  | n/a |
| EBV, CMV |  | Negative |  | n/a |
| LTT BCG | 1.9 (>2.5) |  |  |  |
| LTT Candida Albicans | 1.5 (>2.5) |  |  |  |
| LTT phytohemagglutinin | 4.9 (>2.5) |  |  |  |
|  |  |  |  |  |
| Evaluation 2 | **P1** | **P2** | **P3** | **P4** |
| Age at evaluation (years) | 3 years | 6 years & 2 months |  |  |
| T lymphocytes (number and percentage) | 5792, 62.5% | 1830, 75% |  |  |
| B lymphocytes (number and percentage) | 2029, 21.9% | 160, 7% |  |  |
| NK cells (number and percentage) | 890, 9.7% | 200, 8% |  |  |
| IgG (g/L) | 3.82* (On IVIG) | 4.3 |  |  |
| IgM (g/L) | 0.27 | 0.41 |  |  |
| IgA (g/L) | 0.05 | 0.93 |  |  |
| IgE (IU/mL) | 0.6 | Not done |  |  |
| DHR | NA | Normal |  |  |
| EBV, CMV | Negative | Negative |  |  |
