# Supplementary material for: Complete and partial forms of X-linked MCTS1 deficiency in patients with mycobacterial disease
Source: J Hum Immun. 2026 Jan 30;2(2):e20250073. doi: 10.70962/jhi.20250073 (PMC12857535; doi:10.70962/jhi.20250073)
Supplement: Table S5 — shows the overlap in variants between P1 and P3. [file jhi_20250073_tables5.docx]

**Table S5:** Overlap in variants between P1 and P3.

| **Position(hg38)** | **SNP** | **Reference** | **P1(from Saudi Arabia)** | **P3(from China)** | **Frequency in the general population** |
| --- | --- | --- | --- | --- | --- |
| 119649918 | rs6603539 | **G** |  | **A** |  |
| 119653059 | rs5957203 | **A** |  | **T** |  |
| 119693354 | rs17261138 | **A** | **C** |  |  |
| 119695516 | rs2873171 | **G** |  |  |  |
| 119758925 | rs2782222 | **G** | **C** | **C** |  |
| 119759091 | rs2782223 | **C** | **A** | **A** |  |
| 119759174 | rs2782224 | **T** | **C** | **C** |  |
| 119759450 | rs2782225 | **T** | **C** | **C** |  |
| 119786846 | rs7885997 | **C** | **T** |  |  |
| 119835112 | rs2239963 | **A** | **C** |  |  |
| 119840544 | rs2285552 | **A** | **C** |  |  |
| 119843369 | rs2496213 | **A** | **G** |  |  |
| 119851635 | rs2428212 | **C** | **T** |  |  |
| 119853214 | rs2496219 | **T** |  |  |  |
| 119870442 | rs1858934 | **T** | **A** |  |  |
| 119871742 | rs708463 | **C** | **G** | **G** |  |
| 119920038 | rs3829719 | **C** | **T** |  |  |
| 119924811 | rs194294 | **C** |  |  |  |
| 119930556 | rs194299 | **C** |  |  |  |
| 119931930 | rs194302 | **G** | **A** | **A** |  |
| 119932623 | rs194303 | **G** | **C** |  |  |
| 119936448 | rs194307 | **T** | **A** | **A** | 56% |
| 119936604 | rs194308 | **A** | **T** | **T** | 56% |
| 119937840 | rs11797947 | **C** | **T** |  |  |
| 120015056 | rs17327277 | **T** | **C** |  |  |
| 120075703 | rs3764827 | **A** | **C** |  |  |
| 120115974 | rs6646572 | **G** |  |  |  |
| 120115980 | rs6646573 | **G** |  |  |  |
| 120256111 | rs7882480 | **C** | **T** |  |  |
| 120256610 | rs11537755 | **A** | **G** |  |  |
| 120258025 | rs1044413 | **G** | **A** |  |  |
| 120258941 | rs5910811 | **G** | **A** |  |  |
| 120287312 | rs45453700 | **C** |  |  |  |
| 120311160 | rs3810749 | **C** | **T** |  |  |
| 120366604 | rs2072452 | **T** |  | **C** |  |
| 120376289 | rs3213723 | **C** |  |  |  |
| 120437218 | rs14410 | **G** |  |  |  |
| 120437686 | rs42889 | **T** | **C** | **C** | 63% |
| 120456678 | rs12097 | **T** |  |  |  |
| **120605559** | **c.164+1_164+4GTAAdel** |  |  |  |  |
| 120733466 | rs5909752 | **C** | **G** |  |  |
| 120875125 | rs871733 | **T** |  |  |  |
| 121204228 | rs6648925 | **G** | **A** |  |  |
| 121371069 | rs5956321 | **A** | **G** |  |  |
| 121371215 | rs7885213 | **G** | **T** |  |  |
| 121864081 | rs883386 | **G** | **A** |  |  |
| 123117143 | rs6648942 | **G** |  |  |  |
| 123117866 | rs6648485 | **A** |  |  |  |
| 123202596 | rs2285127 | **T** |  |  |  |
| 123428297 | rs637574 | **C** | **T** |  |  |
| 123514603 | rs3810668 | **T** | **C** |  |  |
| 123558550 | rs2851732 | **G** | **T** |  |  |
| 123601370 | rs2473181 | **G** | **C** |  |  |
| 123624678 | rs2072914 | **G** | **A** | **A** | 32% |
| 123628116 | rs2498051 | **T** | **C** |  |  |
| 123631899 | rs3764750 | **T** | **C** |  |  |
| 123632692 | rs3765260 | **T** | **A** |  |  |
| 123671570 | rs2105889 | **C** | **A** |  |  |
| 124063707 | rs5911742 | **T** | **C** |  |  |
| 124173566 | rs5911783 | **A** | **G** |  |  |
| 124173844 | rs2279726 | **C** | **T** |  |  |
| 124174180 | rs2279725 | **A** | **G** |  |  |
| 124332689 | rs6649195 | **G** | **C** |  |  |
| 124372254 | rs7876065 | **T** | **A** |  |  |
| 124379254 | rs5958476 | **T** | **G** |  |  |
| 124380156 | rs6648597 | **G** |  | **A** |  |
| 124453260 | rs2223416 | **C** | **A** |  |  |
| 124503898 | rs2051578 | **G** | **T** |  |  |
| 125322550 | rs3135237 | **A** | **G** |  |  |
| 125322845 | rs3135238 | **T** | **C** |  |  |
| 125326737 | rs956367 | **C** | **T** |  |  |
| 125327122 | rs1970784 | **A** | **G** |  |  |
| 125327302 | rs1970785 | **T** | **C** |  |  |
| 125327322 | rs1970786 | **T** | **C** |  |  |
| 125327619 | rs1970787 | **C** | **T** |  |  |
| 125327740 | rs1970788 | **C** | **T** |  |  |
| 126164707 | rs3761552 | **G** |  |  |  |
| 126165205 | rs12014937 | **T** |  |  |  |
| 126165484 | rs10126452 | **G** |  |  |  |
| 126166153 | rs7880872 | **C** |  |  |  |
| 126819635 | rs12849661 | **C** | **A** |  |  |
| 126819949 | rs5931715 | **A** | **G** |  |  |
| 126821445 | rs2269777 | **G** |  | **A** |  |
| 126821661 | rs2269776 | **A** |  | **T** |  |
| 126821789 | rs2269775 | **C** |  | **T** |  |
| 129042586 | rs4830110 | **T** | **A** |  |  |
| 129493182 | rs12835315 | **A** |  |  |  |
| 129493325 | rs12841689 | **T** |  |  |  |
| 129545053 | rs5977104 | **C** |  | **G** |  |
| 129560490 | rs3761583 | **A** |  |  |  |
| 129588307 | rs1159042 | **A** |  |  |  |
| 129591880 | rs6529391 | **A** |  |  |  |
| 129647539 | rs3115759 | **C** |  |  |  |
| 129647887 | rs3115758 | **C** |  |  |  |
| 129648435 | rs3115757 | **C** |  |  |  |
| 129648559 | rs5977126 | **G** |  |  |  |
| 129654737 | rs2281069 | **A** |  | **G** |  |
| 129746638 | rs3747343 | **T** |  |  |  |
| 129751591 | rs373374303 | **A** | **G** |  |  |
| 129751595 | rs5932673 | **A** | **G** |  |  |
| 129759441 | rs2076205 | **C** |  |  |  |
| 129793914 | rs859577 | **T** | **C** | **C** | 52% |
| 129794563 | rs112929447 | **C** |  |  |  |
| 129804982 | rs1053486 | **T** | **C** |  |  |
| 129814917 | rs5932686 | **T** | **C** |  |  |
| 129823664 | rs2275605 | **T** | **C** |  |  |
| 129980472 | rs186500011 | **T** | **G** |  |  |
| 129980473 | rs189716956 | **T** | **C** |  |  |
| 130013103 | rs4830173 | **T** |  | **C** |  |
| 130052037 | rs12388749 | **C** | **T** |  |  |
| 130065837 | rs3788848 | **T** | **C** |  |  |
| 130065841 | rs3788847 | **C** | **T** |  |  |
| 130066326 | rs3788845 | **C** |  | **G** |  |
| 130067204 | rs2181440 | **C** | **T** | **T** |  |
| 130110179 | rs210013 | **T** |  |  |  |
| 130110199 | rs210014 | **T** |  |  |  |
| 130110286 | rs210015 | **C** | **G** |  |  |
| 130110293 | rs210016 | **C** | **G** |  |  |
| 130110577 | rs210017 | **T** |  |  |  |
| 130110858 | rs210018 | **A** | **C** |  |  |
| 130110893 | rs201372991 | **G** |  |  |  |
| 130110924 | rs210019 | **A** |  |  |  |
| 130136005 | rs209998 | **G** | **A** |  |  |
| 130149545 | rs1139851 | **A** | **G** |  |  |
| 130156725 | rs189994 | **T** | **C** |  |  |
| 130203580 | rs592368 | **G** | **A** |  |  |
| 130203857 | rs593323 | **C** | **T** |  |  |
| 130204792 | rs608117 | **G** | **A** |  |  |
| 130215321 | rs209238 | **T** | **G** |  |  |
| 130220266 | rs1160681 | **C** | **A** |  |  |
| 131083046 | rs5932835 | **G** | **C** |  |  |
| 131089163 | rs3810727 | **G** | **A** | **A** |  |
| 131089786 | rs3788 | **C** | **T** |  |  |
| 131274771 | rs6529473 | **G** |  | **A** |  |
| 131278204 | rs4524963 | **C** |  |  |  |
| 131279468 | rs10521765 | **G** | **C** |  |  |
| 131281844 | rs1128617 | **T** |  |  |  |
| 131282984 | rs5932877 | **C** | **T** | **T** |  |
| 131285672 | rs4830221 | **A** | **G** |  |  |
| 131286032 | rs5930458 | **T** | **C** | **C** |  |
| 131286441 | rs4495600 | **T** | **C** | **C** |  |
| 131289385 | rs6637829 | **C** |  |  |  |
| 131289569 | rs6637830 | **G** | **C** |  |  |
| 131544493 | rs499030 | **A** | **G** | **G** |  |
| 131544622 | rs17316625 | **C** | **G** |  |  |
| 131544870 | rs655415 | **A** | **C** | **C** |  |
