# Supplementary material for: Complete and partial forms of X-linked MCTS1 deficiency in patients with mycobacterial disease
Source: J Hum Immun. 2026 Jan 30;2(2):e20250073. doi: 10.70962/jhi.20250073 (PMC12857535; doi:10.70962/jhi.20250073)
Supplement: Table S6 — shows the homozygous variants and heterozygous pLOF variants present in P1. [file jhi_20250073_tables6.docx]

**Table S6:** Homozygous variants and heterozygous pLOF variants present in P1.

| **Chr** | **POS** | **Zygo** | **Ref\|SampDp** | **Alt\|TotalDp** | **Consequence** | **SYMBOL** | **HGVSp** | **gnomAD_AF** | **CADD_phred** |
| --- | --- | --- | --- | --- | --- | --- | --- | --- | --- |
| chr1 | 175129924 | hom | CCTTCTTCTT | C | inframe_deletion | KIAA0040 | p.Lys73_Lys75del | 7.55E-05 | . |
| chr4 | 17818976 | hom | G | A | missense_variant | NCAPG | p.Val290Ile | 7.56E-05 | 26.7 |
| chr4 | 44685281 | hom | G | T | missense_variant | GUF1 | p.Arg205Ser | - | 23 |
| chrX | 119746082 | hom | T | G | stop_gained | MCTS1 | p.Leu171Ter | - | 41 |
| chr1 | 1.52E+08 | het | AAT | A | frameshift_variant | HRNR | p.Tyr2810PhefsTer8 | 1.19E-05 | . |
| chr1 | 2.49E+08 | het | C | CT | frameshift_variant | OR2T2 | p.Tyr279LeufsTer18 | - | . |
| chr10 | 1.27E+08 | het | AC | A | frameshift_variant | CTBP2 | p.Val597TrpfsTer17 | - | . |
| chr11 | 1018229 | het | CGAGGTAGGTGTTTTGTT | C | frameshift_variant | MUC6 | p.Asn1519AlafsTer12 | - | . |
| chr11 | 89880500 | het | C | A | missense_variant&splice_region | NAALAD2 | p.Ser66Tyr | - | 23.5 |
| chr13 | 28552364 | het | TCGCGCGGCACCGCCGTCCGGTCGCTGAAG | T | frameshift_variant | URAD | p.Phe125AlafsTer92 | - | . |
| chr15 | 40895160 | het | G | GA | frameshift_variant | CASC5 | p.Asn12LysfsTer2 | - | . |
| chr15 | 65489662 | het | G | A | stop_gained | CILP | p.Arg988Ter | 8.36E-05 | 40 |
| chr16 | 14702916 | het | C | T | missense_variant&splice_region | PARN | p.Gly146Arg | 6.15E-05 | 34 |
| chr16 | 58292296 | het | C | T | stop_gained | CCDC113 | p.Arg139Ter | 3.18E-05 | 37 |
| chr16 | 71956377 | het | G | A | missense_variant&splice_region | IST1 | p.Ala185Thr | - | 21.5 |
| chr17 | 6902373 | het | C | A | missense_variant&splice_region | ALOX12 | p.Ala215Asp | - | 25.6 |
| chr19 | 7600462 | het | T | C | missense_variant&splice_region | PNPLA6 | p.Met9Thr | - | 23 |
| chr19 | 51462421 | het | CA | C | frameshift_variant&stop_lost | KLK6 | p.Ter245AspfsTer5 | - | . |
| chr22 | 23047143 | het | GCCCC | G | frameshift_variant | IGLV3-22 | p.Ala61ValfsTer4 | - | . |
| chr3 | 38048443 | het | C | A | stop_gained | VILL | p.Ser823Ter | 4.38E-05 | 47 |
| chr3 | 75714805 | het | TG | T | frameshift_variant | FRG2C | p.Arg156AlafsTer10 | - | . |
| chr3 | 75787348 | het | CCCCTG | C | frameshift_variant | ZNF717 | p.Thr467ArgfsTer5 | - | . |
| chr3 | 75787645 | het | GAA | G | frameshift_variant | ZNF717 | p.Phe369SerfsTer3 | - | . |
| chr4 | 1388753 | het | C | CCG | frameshift_variant | CRIPAK | p.Met153ProfsTer38 | - | . |
| chr4 | 1388755 | het | CAT | C | frameshift_variant | CRIPAK | p.Met153AlafsTer254 | - | . |
| chr5 | 1.51E+08 | het | GT | G | frameshift_variant | SLC36A3 | p.Thr447HisfsTer31 | 7.95E-06 | . |
| chr7 | 80302113 | het | AAACGGCTGCAG | A | frameshift_variant | CD36 | p.Lys385SerfsTer29 | - | . |
| chr9 | 1.36E+08 | het | CG | C | frameshift_variant | CEL | p.Ala623ProfsTer15 | - | . |
