# Supplementary material for: Complete and partial forms of X-linked MCTS1 deficiency in patients with mycobacterial disease
Source: J Hum Immun. 2026 Jan 30;2(2):e20250073. doi: 10.70962/jhi.20250073 (PMC12857535; doi:10.70962/jhi.20250073)
Supplement: Table S7 — shows the homozygous non-synonymous and heterozygous pLOF variants present in P2. [file jhi_20250073_tables7.docx]

**Table S7:** Homozygous non-synonymous and heterozygous pLOF variants present in P2.

| VAR | CHR | POS | REF | ALT | GENE | CONSEQ | ZYGO | AA_CHANGE | gnomADV3_AF | gnomADV3_hom |
| --- | --- | --- | --- | --- | --- | --- | --- | --- | --- | --- |
| 10_37145057_G_A | 10 | 37145057 | G | A | ANKRD30A | Essential_splicing | het |  | 3.29E-05 | 0 |
| 11_93727115_AG_A | 11 | 93727115 | AG | A | CEP295 | INDEL-frameshift | het | Glu2214AsnfsTer19 | |  |
| 12_77024164_TGG_T | 12 | 77024164 | TGG | T | E2F7 | INDEL-frameshift | het | Pro862GlnfsTer9 | |  |
| 13_113811735_G_C | 13 | 113811735 | G | C | TMEM255B | Essential_splicing | het |  | 6.58E-06 | 0 |
| 13_57142529_AC_A | 13 | 57142529 | AC | A | PRR20A | INDEL-frameshift | het | Pro70LeufsTer38 | |  |
| 13_99866380_C_T | 13 | 99866380 | C | T | CLYBL | Stop-Gained | het | Arg259Ter | 0.0227 | 61 |
| 14_22906361_G_A | 14 | 22906361 | G | A | RBM23 | Stop-Gained | het | Arg79Ter | 0.000118 | 0 |
| 15_30937260_G_A | 15 | 30937260 | G | A | FAN1 | Essential_splicing | het |  | 0.0101 | 29 |
| 16_31508283_TCCCGTCCGCGGCGGCGCGGCAGC_T | 16 | 31508283 | TCCCGTCCGCGGCGGCGCGGCAGC | T | RUSF1 | INDEL-frameshift | het | Gly23GlufsTer23 | |  |
| 17_3292309_T_A | 17 | 3292309 | T | A | OR3A1 | Stop-Gained | het | Lys92Ter | 0.0191 | 69 |
| 17_50763441_G_A | 17 | 50763441 | G | A | ANKRD40CL | Stop-Gained | het | Arg53Ter | 0.00984 | 6 |
| 18_62325432_G_A | 18 | 62325432 | G | A | TNFRSF11A | Essential_splicing | het |  | 0.0242 | 48 |
| 18_674316_G_A | 18 | 674316 | G | A | ENOSF1 | Stop-Gained | het | Gln441Ter |  |  |
| 19_21082352_AG_A | 19 | 21082352 | AG | A | ZNF714 | Essential_splicing | het |  | 0.00312 | 1 |
| 19_43486826_CG_C | 19 | 43486826 | CG | C | PHLDB3 | INDEL-frameshift | het | Gly432AlafsTer9 | 5.26E-05 | 0 |
| 19_52582594_C_T | 19 | 52582594 | C | T | ZNF701 | Stop-Gained | het | Arg179Ter | 0.00113 | 0 |
| 19_55294011_TCTCAGCCACAGAGACCTAAAGCCCGA_T | 19 | 55294011 | TCTCAGCCACAGAGACCTAAAGCCCGA | T | BRSK1 | Essential_splicing | het |  |  |  |
| 19_55955615_T_G | 19 | 55955615 | T | G | NLRP8 | Stop-Gained | het | Tyr519Ter |  |  |
| 1_11766203_T_TG | 1 | 11766203 | T | TG | C1orf167 | INDEL-frameshift | het | Ser142LysfsTer37 | 1.97E-05 | 0 |
| 1_228282202_A_T | 1 | 228282202 | A | T | OBSCN | Stop-Gained | het | Arg3252Ter | 0.0224 | 288 |
| 1_230333011_C_CGCGCG | 1 | 230333011 | C | CGCGCG | PGBD5 | INDEL-frameshift | het | Arg369ProfsTer15 | |  |
| 1_241996175_G_T | 1 | 241996175 | G | T | MAP1LC3C | Stop-Gained | het | Cys144Ter |  |  |
| 1_26282398_ACC_A | 1 | 26282398 | ACC | A | UBXN11 | INDEL-frameshift | het | Gly488SerfsTer? | 0.00369 | 0 |
| 1_26282401_GGGAC_G | 1 | 26282401 | GGGAC | G | UBXN11 | INDEL-frameshift | het | Cys486SerfsTer? | 0.00214 | 1 |
| 1_26301689_C_T | 1 | 26301689 | C | T | UBXN11 | Essential_splicing | het |  | 0.000808 | 0 |
| 1_27373179_AG_A | 1 | 27373179 | AG | A | FCN3 | INDEL-frameshift | het | Leu117SerfsTer65 | 0.0184 | 34 |
| 1_46402196_GTGGGAAAGGTAAGGCCAGCCAAGGCCAGCCCCTCCC_G | 1 | 46402196 | GTGGGAAAGGTAAGGCCAGCCAAGGCCAGCCCCTCCC | G | FAAH | Essential_splicing | het |  | 0.00689 | 11 |
| 20_29092559_T_G | 20 | 29092559 | T | G | FRG1DP | Essential_splicing | het |  |  |  |
| 20_30702854_C_T | 20 | 30702854 | C | T | ANKRD20A21P | Essential_splicing | het |  | 0.0372 | 0 |
| 21_32603251_C_T | 21 | 32603251 | C | T | CFAP298 | Stop-Gained | het | Trp192Ter |  |  |
| 2_113006655_AC_A | 2 | 113006655 | AC | A | IL36A | INDEL-frameshift | het | Ile63SerfsTer5 | 6.57E-06 | 0 |
| 2_159349703_C_T | 2 | 159349703 | C | T | BAZ2B | Essential_splicing | het |  | 0.0336 | 175 |
| 2_201492754_CTGTT_C | 2 | 201492754 | CTGTT | C | C2CD6 | INDEL-frameshift | het | Asn1195ValfsTer10 | 0.000579 | 0 |
| 2_233985870_G_A | 2 | 233985870 | G | A | TRPM8 | Essential_splicing | het |  | 2.63E-05 | 0 |
| 2_24164308_GC_G | 2 | 24164308 | GC | G | FAM228B | INDEL-frameshift | het | Gln303ArgfsTer18 | 0.0318 | 101 |
| 2_27129755_A_ATACT | 2 | 27129755 | A | ATACT | ABHD1 | INDEL-frameshift | het | Val209ThrfsTer29 | 0.000118 | 0 |
| 2_37372476_ATG_A | 2 | 37372476 | ATG | A | QPCT | Essential_splicing | het |  | 0.000333 | 0 |
| 2_85619218_A_G | 2 | 85619218 | A | G | USP39 | Essential_splicing | het |  |  |  |
| 3_151447829_TCTTA_T | 3 | 151447829 | TCTTA | T | IGSF10 | INDEL-frameshift | het | Ser716ArgfsTer8 | 0.00212 | 2 |
| 3_98025679_G_T | 3 | 98025679 | G | T | GABRR3 | Stop-Gained | het | Cys42Ter |  |  |
| 4_4147516_C_G | 4 | 4147516 | C | G | UNC93B4 | Essential_splicing | het |  | 0.00802 | 92 |
| 4_87318348_ATCTCT_A | 4 | 87318348 | ATCTCT | A | HSD17B13 | INDEL-frameshift | het | Glu98AspfsTer14 | 0.000677 | 1 |
| 5_119634664_G_T | 5 | 119634664 | G | T | FAM170A | Stop-Gained | het | Glu306Ter |  |  |
| 5_96765327_TAAAAAA_T | 5 | 96765327 | TAAAAAA | T | CAST | Essential_splicing | het |  | 1.01E-05 | 0 |
| 5_96765327_TAAAA_T | 5 | 96765327 | TAAAA | T | CAST | Essential_splicing | het |  | 0.008 | 21 |
| 6_148514458_TAAAAAAAAAAA_T | 6 | 148514458 | TAAAAAAAAAAA | T | SASH1 | Essential_splicing | het |  | 0.00122 | 0 |
| 6_170561966_AGCAGCAGCAGCAGCAGCAGCAG_A | 6 | 170561966 | AGCAGCAGCAGCAGCAGCAGCAG | A | TBP | INDEL-frameshift | het | Gln77HisfsTer60 | 0.00254 | 1 |
| 6_29944621_A_T | 6 | 29944621 | A | T | HLA-A | Essential_splicing | het |  | 0.00112 | 0 |
| 6_31356751_G_GGA | 6 | 31356751 | G | GGA | HLA-B | INDEL-frameshift | het | Gln94SerfsTer58 | 0.0164 | 32 |
| 6_32521970_G_GCC | 6 | 32521970 | G | GCC | HLA-DRB5 | INDEL-frameshift | het | Ala102GlyfsTer28 | 0.0248 | 0 |
| 6_32584184_G_GT | 6 | 32584184 | G | GT | HLA-DRB1 | INDEL-frameshift | het | Gln99ThrfsTer29 | 0.0273 | 100 |
| 6_32584185_C_CT | 6 | 32584185 | C | CT | HLA-DRB1 | INDEL-frameshift | het | Gln99AlafsTer29 | 0.0264 | 101 |
| 6_75151862_C_T | 6 | 75151862 | C | T | COL12A1 | Essential_splicing | het |  | 0.00333 | 4 |
| 6_80011806_G_C | 6 | 80011806 | G | C | TTK | Essential_splicing | het |  | 0.047 | 563 |
| 7_19698409_CT_C | 7 | 19698409 | CT | C | POLR1F | INDEL-frameshift | het | Lys308ArgfsTer18 | 0.000324 | 0 |
| 7_30850557_T_G | 7 | 30850557 | T | G | MINDY4 | Essential_splicing | het |  | 2.63E-05 | 0 |
| 9_127897960_T_TG | 9 | 127897960 | T | TG | ST6GALNAC6 | INDEL-frameshift | het | Ser8GlnfsTer4 | 0.00027 | 0 |
| 9_129868454_C_T | 9 | 129868454 | C | T | USP20 | Essential_splicing | het |  | 0.0128 | 20 |
| 9_133071565_GC_G | 9 | 133071565 | GC | G | CEL | INDEL-frameshift | het | Ala689ProfsTer15 | 0 | 0 |
| X_1294453_C_CACAGAAAGGTCGGTGAG | X | 1294453 | C | CACAGAAAGGTCGGTGAG | CSF2RA | INDEL-frameshift | het |  | 0.0114 | 30 |
| 10_46123271_A_G | 10 | 46123271 | A | G | AGAP7P | Essential_splicing | hom |  | 0.00175 | 1 |
| 14_64137874_C_G | 14 | 64137874 | C | G | SYNE2 | Missense | hom | Pro4912Ala | 0.0383 | 147 |
| 19_39902492_C_T | 19 | 39902492 | C | T | FCGBP | Missense | hom | Glu1446Lys | 4.04E-05 | 0 |
| 19_39902495_C_G | 19 | 39902495 | C | G | FCGBP | Missense | hom | Glu1445Gln | 0 | 0 |
| 19_39902509_C_T | 19 | 39902509 | C | T | FCGBP | Missense | hom | Gly1440Asp | 0.000172 | 0 |
| 1_13308378_G_A | 1 | 13308378 | G | A | PRAMEF33 | Missense | hom | Ala306Thr | 0.0103 | 14 |
| 1_152213383_C_T | 1 | 152213383 | C | T | HRNR | Missense | hom | Arg2749His | 3.69E-05 | 1 |
| 1_152213385_G_C | 1 | 152213385 | G | C | HRNR | Missense | hom | His2748Gln | 3.65E-05 | 1 |
| 1_217683280_A_G | 1 | 217683280 | A | G | SPATA17 | Missense | hom | Tyr105Cys | 0.00957 | 25 |
| 21_44592338_C_CGGGGCGCAGCAGCTG | 21 | 44592338 | C | CGGGGCGCAGCAGCTG | KRTAP10-6 | INDEL-inframe | hom | Pro49_Ala50insSerCysCysAlaPro | 0.000772 | 0 |
| 6_152364955_G_T | 6 | 152364955 | G | T | SYNE1 | Missense | hom | Ser3346Tyr | 0.00386 | 1 |
| 6_31026506_G_A | 6 | 31026506 | G | A | MUC22 | Missense | hom | Val359Ile | 0.0373 | 489 |
| 6_32584174_GCC_G | 6 | 32584174 | GCC | G | HLA-DRB1 | INDEL-frameshift | hom | Ala102ArgfsTer25 | 0.00194 | 0 |
| 6_32664912_C_T | 6 | 32664912 | C | T | HLA-DQB1 | Missense | hom | Asp89Asn | 0.0284 | 497 |
| 9_34726824_A_C | 9 | 34726824 | A | C | FAM205A | Missense | hom | Leu139Arg | 0.0116 | 23 |
| X_119758952_G_C | X | 119758952 | G | C | SOWAHD | Missense | hom | Glu95Asp |  |  |
| X_136556533_T_A | X | 136556533 | T | A | VGLL1 | Missense | hom | His257Gln | 0.000937 | 1 |
| X_153319501_G_A | X | 153319501 | G | A | PNMA6F | Missense | hom | Arg392Cys | 0.00353 | 1 |
| X_8466313_T_C | X | 8466313 | T | C | VCX3B | Missense | hom | Leu224Pro | 0.00977 | 10 |
