# Supplementary material for: Complete and partial forms of X-linked MCTS1 deficiency in patients with mycobacterial disease
Source: J Hum Immun. 2026 Jan 30;2(2):e20250073. doi: 10.70962/jhi.20250073 (PMC12857535; doi:10.70962/jhi.20250073)
Supplement: Table S8 — shows the additional candidate variants present in P3. [file jhi_20250073_tables8.docx]

**Table S8:** Additional candidate variants present in P3.

| Gene | Chromosomal location | Mutation information | Zygote type | Source of variants |
| --- | --- | --- | --- | --- |
| ATP8B1 | Chr18：55359108 | NM_005603:exon12:  c.1151G>A(p.R384H) | Het | paternal |
| ATP8B1 | chr18:  55317626 | NM_005603:exon27:  c.3504G>A(p.M1168I) | Het | Maternal |
| EARS2 | chr16:23556001 | NM_001083614:exon3:  c.319C>T(p.R107C） | Het | Maternal |
| PEX1 | Chr7:  92148393 | NM_000466:exon3:  c.274-1G>A | Het | Maternal |
| SLC22A5 | chr5:  131728257 | NM_003060:exon8:  c.1400C>G(p.S467C) | Het | Maternal |
| UNC13D | chr17:  73824885 | NM_199242:exon31:  c.3134C>T(p.T1045M) | Het | Maternal |
| CACNA1F | chrX:49067864 | NM_005183:exon36:  c.4211T>C(p.F1404S) | Hemi | Maternal |
| IRS4 | chrX:107976495 | NM_003604:exon1:  c.3080C>T(p.P1027L) | Hemi | Maternal |
| COG5 | chr7:106888868 | NM_006348:exon17:  c.1826T>C(p.I609T) | Hom | Maternal/Paternal |
